# Quercetin influences intestinal dysbacteriosis and delays alveolar epithelial cell senescence by regulating PTEN/PI3K/AKT signaling in pulmonary fibrosis

**DOI:** 10.1007/s00210-023-02913-8

**Published:** 2023-12-28

**Authors:** Wenjuan Wu, Xinhui Wu, Lingxiao Qiu, Ruijie Wan, Xiaoming Zhu, Song Chen, Xinying Yang, Xueya Liu, Jizhen Wu

**Affiliations:** 1 Department of Geriatric Medicine, Henan Provincial People’s Hospital, Zhengzhou University, Jinshui District, No. 7 Weiwu Road, Zhengzhou, 450000 Henan China; 2 Department of Traditional Chinese Medicine, Zhengzhou Shuqing Medical College, Zhengzhou, 450000 Henan China; 3 Department of Respiratory and Critical Care Medicine, The Second Affiliated Hospital of Army Medical University, Chongqing, 400037 China; 4 Department of Thoracic Surgery, Henan Provincial People’s Hospital, Zhengzhou University, Zhengzhou, 450000 Henan China; 5 Translational Research Institute, Henan Provincial People’s Hospital, Zhengzhou University, Zhengzhou, 450000 Henan China

**Keywords:** Quercetin, Pulmonary fibrosis, Cellular senescence, PTEN

## Abstract

Pulmonary fibrosis is a chronic and progressive lung disease with high mortality. This study aims to explore the protective mechanism of quercetin against pulmonary fibrosis regarding cell senescence and gut microbiota. Rats were intratracheally injected with bleomycin (BLM) to establish a pulmonary fibrosis rat model. RLE-6TN cells were stimulated with BLM to build the model of alveolar epithelial cell senescence, and RLE-6TN-derived conditional medium (CM) was harvested to further culture fibroblasts. Histopathological changes were assessed by H&E and Masson staining. α-SMA expression was assessed by immunofluorescence assay. Senescence-associated β-galactosidase (SA-β-gal) staining and senescence-associated secretory phenotype (SASP) cytokine assay were conducted to assess cellular senescence. Gut microbiota was analyzed by 16S rRNA gene sequencing. The fibrosis-, senescence-, and PTEN/PI3K/AKT signaling–related proteins were examined by western blot. In BLM-induced pulmonary fibrosis rats, quercetin exerted its protective effects by reducing histological injury and collagen deposition, lessening cellular senescence, and regulating gut microbiota. In BLM-induced alveolar epithelial cell senescence, quercetin inhibited senescence, lessened SASP cytokine secretion of alveolar epithelial cells, and further ameliorated collagen deposition in fibroblasts. In addition, quercetin might exert its functional effects by regulating the PTEN/PI3K/AKT signaling pathway. Moreover, quercetin regulated intestinal dysbacteriosis in BLM-induced pulmonary fibrosis rats, especially boosting the abundance of *Akkermansia*. To conclude, our findings provide an in-depth understanding of the potential mechanism behind the protective role of quercetin against pulmonary fibrosis.

## Introduction

Pulmonary fibrosis is a chronic and progressive interstitial lung disease of unknown origin, characterized by remodeling of the interstitium, distal airway, and alveolar spaces, and clinically manifested as dry cough, fatigue, and exertional dyspnea (Glass et al. 2022). Pulmonary fibrosis has a poor prognosis, with a median survival time of only 2.5–3.5 years after diagnosis, if untreated (Raghu et al. 2011; Strongman et al. 2018; Ley et al. 2011). Epidemiological investigation and clinical observations indicate that pulmonary fibrosis is an age-related disorder, with the incidence and mortality increasing dramatically with age. A recent study from the UK cohort revealed that approximately 85% of patients with pulmonary fibrosis were older than 70 years old when first diagnosed. With the rapid rise in the aging population worldwide, the socio-economic burden of pulmonary fibrosis is expected to steadily grow in the future (Spagnolo et al. 2021; Diamantopoulos et al. 2018).

Despite that the comprehensive understanding of pulmonary fibrosis pathogenesis remains elusive, cell senescence is one of the most important biological functions in pulmonary fibrosis pathogenesis. Although senescence is a state of irreversible cell cycle arrest, senescent cells still remain metabolically active and secrete various cytokines, chemokines, growth factors, and proteases, termed the senescence-associated secretory phenotype (SASP) (McHugh and Gil 2018; Salama et al. 2014). A growing body of evidence supports a correlation between pulmonary fibrosis pathogenesis and cellular senescence (Kellogg et al. 2021). Cellular senescence has been discovered in lung fibroblasts and alveolar epithelial cells, and the senescent fibroblasts produced exaggerated amounts of SASP, exacerbating the progression of pulmonary fibrosis (Mora et al. 2017; Alvarez et al. 2017). It was observed from the lung biopsies of patients with pulmonary fibrosis that the expression of senescence-associated β-galactosidase (SA-β-gal), a classical hallmark of cellular senescence, was aberrantly elevated (Kellogg et al. 2021). In addition, recent reports have identified pro-fibrotic factors in the SASP, indicating that senescent cells are crucial drivers of pulmonary fibrosis (Merkt et al. 2020).

Quercetin, ubiquitously present in fruits and vegetables, is the major representative of the flavonoid subclass of flavonols and has multiple bio-activities including anti-inflammatory, anti-microbial, anti-oxidant, anti-hyperglycemia, anti-hyperlipidemia, anti-cancer, cardio-protective, and neuro-protective effects (Hosseini et al. 2021; Singh et al. 2021). Meanwhile, the protective role of quercetin against pulmonary fibrosis has been evidenced in previous studies. In bleomycin (BLM)-induced lung fibrosis in rats, oral administration of quercetin significantly reduced collagen deposition and blocked the production of pro-fibrotic mediators, serving as a promising candidate drug for the treatment of pulmonary fibrosis (Verma et al. 2013; Takano et al. 2020; Boots et al. 2020). Of note, quercetin could lessen the secretion of SASP in BLM-mediated fibroblasts in vitro and BLM-induced aged mice in vivo, highlighting the effectiveness of quercetin on senescence and suggesting quercetin as a potential senolytic therapy for pulmonary fibrosis treatment (Lim et al. 2015; Sellares and Rojas 2019; Hohmann et al. 2019); however, except fibroblasts, whether quercetin regulates senescence in alveolar epithelial cells needs to be verified. Additionally, microbial imbalance or dysbiosis in the gut microbiome is usually linked to aberrant inflammation and immune responses, which ultimately facilitate the progression of respiratory diseases, including pulmonary fibrosis. Gut microbiota–dependent metabolites have been evidenced to deteriorate vascular aging and endothelial cell senescence (Ke et al. 2018), disclosing the regulation of gut microbiota on cellular senescence.

Based on the findings above, the purpose of this study is to explore the protective mechanism of quercetin against pulmonary fibrosis regarding cell senescence and gut microbiota. Therefore, this study provides a novel perspective on the regulatory mechanism of quercetin regarding the prevention of pulmonary fibrosis.

## Materials and methods

### Establishment of BLM-induced pulmonary fibrosis rat model and drug treatment

A total of 20 8-week-old female Wistar rats were obtained from Vital River Laboratory Animal Technology Co., Ltd. (Beijing, China) and housed under a standard environment (temperature: 22 ± 2 °C, humidity: 55 ± 5%, 12/12-h light/dark cycle) with free access to water and food. After acclimation for 1 week, all rats were randomly assigned into 4 groups (*n* = 5): control, BLM, BLM+quercetin (75 mg/kg), and BLM+quercetin (100 mg/kg) groups. The pulmonary fibrosis animal model was constructed by an intratracheal injection of 7.5 IU/kg BLM sulfate (Nippon Kayaku, Tokyo, Japan) dissolved in normal saline on day 7 as previously described (Kalantar et al. 2021). The rats in the control group received the same volume of normal saline. Rats in the BLM+quercetin (75 mg/kg) and BLM+quercetin (100 mg/kg) groups were orally administrated with 75 mg/kg and 100 mg/kg of quercetin (Sigma-Aldrich, St. Louis, MO, USA), respectively, from day 1 to day 28. All experimental protocols were approved by the Ethics Committee of Henan Provincial People’s Hospital. About 24 h after the last administration, the rats were anesthetized with 50 mg/kg pentobarbital sodium and sacrificed by decapitation. Afterward, the lung tissues were harvested for subsequent analysis. All animal experiments were approved by the Ethics Committee of Henan Provincial People’s Hospital.

### Histopathological examination

The samples of lung tissues were harvested and immersed in 4% paraformaldehyde for 48 h, followed by paraffin embedding and cutting into 4-μm-thick sections. Thereafter, the sections were stained with hematoxylin and eosin (H&E) for observation of histological injury or stained with Masson’s trichrome dye for observation of lung fibrosis. All images were obtained under a light microscope (Olympus Corporation, Tokyo, Japan).

### Immunofluorescence staining

The fixed lung tissues were blocked with 3% BSA for 30 min and exposed to the anti-α-SMA antibody at 4 °C overnight. After washing, the tissues were incubated with Alexa Fluor 488-conjugated anti-IgG secondary antibody at room temperature for 2 h. DAPI was used to stain nuclei, and coverslips were mounted. The fluorescent images were captured under a fluorescence microscope (Olympus Corporation).

### Hydroxyproline (HYP) detection

The lung tissues were homogenized on ice and then hydrolyzed at 100 °C for 20 min. After adjustment of PH value and adsorption of insoluble matter with active carbon, the concentration of HYP was determined by the reaction of oxidized hydroxyproline with 4-(dimethylamino)benzaldehyde (DMAB) at 60 °C for 15 min according to the instructions of Hydroxyproline Assay Kit (Nanjing Jiancheng Bioengineering Institute, Nanjing, China). Finally, the absorbance at 550 nm was detected, and the HYP content was calculated.

### Western blot

Total proteins from lung tissues were exacted by adopting RIPA lysis buffer (Beyotime, Shanghai, China). After quantification of protein concentration dependent on BCA assay (Pierce, Rockford, USA), the same amounts of proteins (30 μg/lane) were separated by 12% SDS-PAGE gel and transferred onto PVDF membranes. Afterward, the membranes were blocked with 5% non-fat milk for 2 h and then probed with primary antibodies at 4 °C overnight. On the following day, the membranes were washed and incubated with HRP-conjugated secondary antibodies for 2 h. The blots were visualized using the ECL detection system (Amersham Pharmacia Biotech, Amersham, UK) and then quantified using the ImageJ software (National Institutes of Health, USA).

### 16S rRNA gene sequencing and analysis

Microbial community genomic DNA was extracted from fresh fecal samples using a DNA Extraction Kit (Qiagen, German). After verifying the quality and quantity of extracted DNA through agarose gel electrophoresis, the hypervariable regions of V3-V4 were amplified by polymerase chain reaction (PCR) using universal primers 341F (5′-CCTACGGGNGGCWGCAG-3′) and 805R (5′-GACTACHVGGGTATCTAATCC-3′). The PCR products were purified using the Qiagen Gel Extraction Kit (Qiagen). Thereafter, high-throughput sequencing was conducted using the Illumina Miseq system with TruSeq Nano DNA LT Library Prep Kit (Illumina MiSeq, USA), and the sequencing library was established.

### Cell culture and treatment

Rat type II alveolar epithelial cell line (RLE-6TN) was purchased from the American Type Culture Collection (ATCC; Manassas, USA), and rat embryonic fibroblast CCC-REPF-1 was obtained from the Chinese Academy of Medical Sciences (Beijing, China).

All cells were cultured in Dulbecco’s Modified Eagle’s Medium (DMEM; Invitrogen, USA) supplemented with 10% fetal bovine serum (FBS; Gibco, USA) under 5% CO_2_ atmosphere at 37 °C.

To build the model of alveolar epithelial cell senescence, RLE-6TN cells were stimulated with different concentrations of BLM (0, 2.5, and 5 μM) for 72 h as previously described (Zhang et al. 2020). For treatment, RLE-6TN cells were pre-treated with quercetin (5, 25, and 50 μM) for 1 h prior to BLM stimulation.

In addition, to simulate alveolar epithelial cell senescence–secreted SASP and observe its effects on lung fibroblast cells, RLE-6TN-derived conditional medium (CM) was harvested to further culture CCC-REPF-1 cells for 48 h.

### Cell transfection

To explore the regulatory mechanism of quercetin on PTEN, the short hairpin RNA (shRNA) targeting PETN (sh-PTEN-1/2/3), as well as its negative control (sh-NC), was designed and synthesized by GenePharma (Shanghai, China). RLE-6TN cells were transfected with the above plasmids adopting Lipofectamine 3000 reagent (Invitrogen) strictly in accordance with the manufacturer’s instructions. Forty-eight hours post transfections, the transfection efficacy was determined by detecting PTEN expression using western blot and quantitative real-time PCR (qRT-PCR) assays.

### qRT-PCR assay

Total RNA from cells was extracted using TRIzol reagent (Invitrogen), followed by reverse transcription into complementary DNA (cDNA) adopting PrimeScript RT Master Mix Kit (TaKaRa, Japan). Thereafter, qPCR reaction was carried out using SYBR Premix Ex Taq Kit (TaKaRa) on a 7500 Fast Real-time PCR System (Applied Biosystems, USA). The mRNA level of PTEN was calculated using the 2^−△△Ct^ method, and β-actin served as the endogenous gene.

### Cell viability assay

Cell viability was assessed by Cell Counting Kit-8 (CCK-8) assay. In brief, RLE-6TN cells were seeded into 96-well plates for culture. After incubation with BLM or quercetin for 72 h, 10 μl of CCK-8 solution (Dojindo Laboratories, Tokyo, Japan) was added to each well, and the plates were incubated at 37 °C for 2 h. Thereafter, the absorbance at 450 nm was measured under a microplate reader.

### Measurement of cytokines

After indicated treatment, the supernatant of the culture medium was collected, and the concentrations of interleukin (IL)-1β, IL-6, IL-8, and tumor necrosis factor-alpha (TNF-α) in the supernatant were measured using their corresponding enzyme-linked immunosorbent assay (ELISA) kits from Solarbio (Beijing, China). The absorbance at 450 nm was read under a microplate reader.

### SA-β-gal staining

Senescence was determined using a SA-β-gal staining kit (Beyotime). In brief, the frozen lung tissues or cells were fixed with 4% formaldehyde for 10–15 min at room temperature and then stained with SA-β-gal solution at 37 °C in line with the manufacturer’s guidelines. After washing, the SA-β-gal-positive images were captured by a light microscope (Olympus Corporation). The SA-β-gal-positive area of cells in each group was quantified using ImageJ software (National Institutes of Health, USA).

### Statistical analysis

All data were analyzed using GraphPad Prism 8.0 (GraphPad Software, Inc.) and presented as mean ± standard deviation (SD) from at least three independent experiments in vitro or groups of 5 rats each in vivo. For multiple comparisons, one-way ANOVA followed by Tukey’s post hoc test was utilized to analyze the differences. The differences were regarded as statistically significant when the *p* value was less than 0.05.

## Results

### Quercetin attenuates pulmonary fibrogenesis and cell senescence and regulates PETN/PI3K/AKT signaling in BLM-induced pulmonary fibrosis rats

**Fig. 1:**
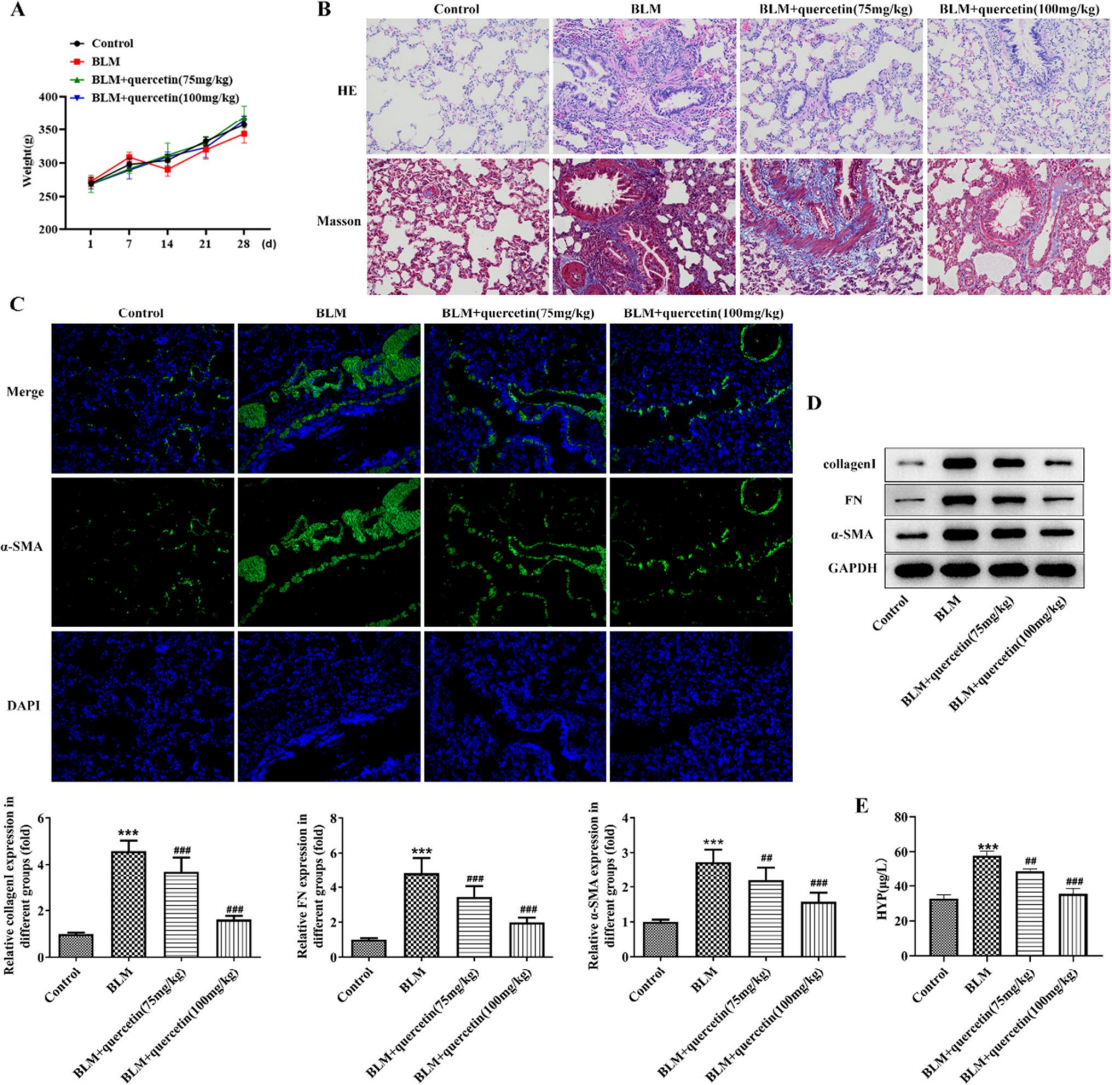
Quercetin attenuates pulmonary fibrogenesis in BLM-induced pulmonary fibrosis rats. The pulmonary fibrosis animal model was constructed by intratracheal injection of BLM in rats, and rats were administrated with quercetin (75 mg/kg and 100 mg/kg). **A** Body weight was monitored every week. **B** H&E staining and Masson staining for histopathological changes in rat lung. **C** Immunofluorescence staining for α-SMA level. **D** Western blot assesses the protein expression of collagen I, FN, and α-SMA in lung tissues. **E** HYP content was measured to reflect the severity degree of fibrosis. ***p < 0.001 vs. control; ##*p* < 0.01 and ###*p* < 0.001 vs. BLM

Firstly, we confirmed the anti-fibrotic effects of quercetin on BLM-induced pulmonary fibrosis rats in the present study. It was observed from Fig. 1A that the body weight of rats exhibited an inflection point following BLM injection in the BLM group while maintaining a steady increase in other groups. Meanwhile, the loosely distributed cellular nodules and destroyed lung parenchymal structure, as well as abundant collagen deposition, were observed in the BLM group, compared to the control group, demonstrating severe pulmonary injury and fibrosis in BLM-induced rats; however, these severe histopathological changes were remarkably attenuated by quercetin administration (Fig. 1B). Pulmonary fibrosis is characterized by ongoing tissue remodeling with accumulation of α-SMA-expressing myofibroblasts within fibroblastic foci. Immunofluorescence staining showed that α-SMA level was remarkably enhanced in BLM-induced rats, which was partly weakened by quercetin administration (Fig. 1C). Consistent with this observation, the protein expression of collagen I, fibronectin (FN), and α-SMA in lung tissues was hugely elevated after BLM treatment, which was obviously hindered following quercetin administration at a concentration-dependent manner (Fig. 1D). HYP is the unique amino acid of collagen; thus, HYP content reflects the severity degree of fibrosis. As expected, we found an elevated HYP content in BLM-induced rats, and quercetin administration greatly restricted HYP content concentration dependently (Fig. 1E).

**Fig. 2:**
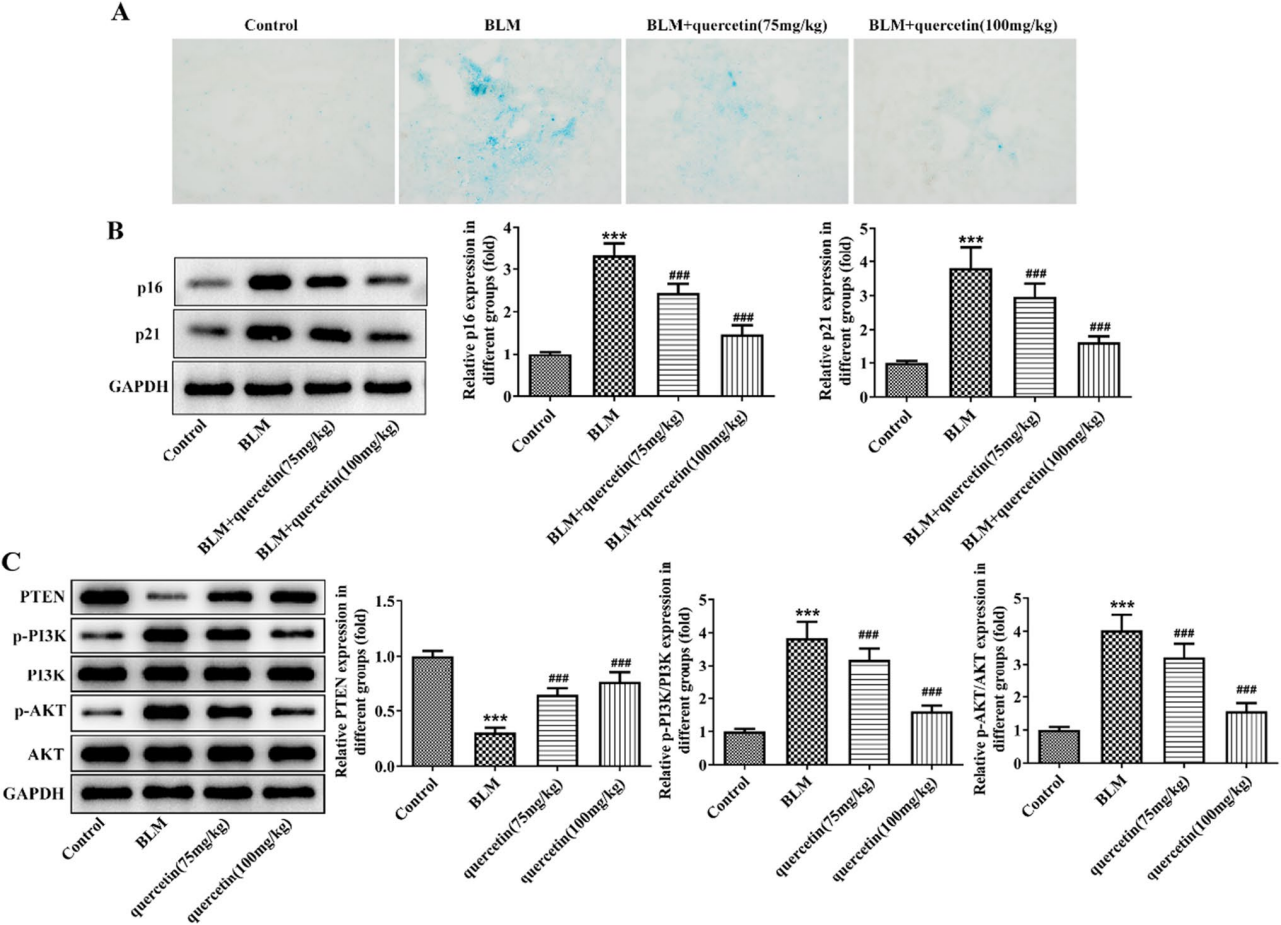
Quercetin attenuates cell senescence and regulates PETN/PI3K/AKT signaling in BLM-induced pulmonary fibrosis rats. **A** SA-β-gal staining was conducted to evaluate cellular senescence of lung tissues. **B** Western blot assesses the protein expression of p16 and p21 in lung tissues. **C** The protein expression of PTEN, p-PI3K, PI3K, p-AKT, and AKT in lung tissues of rats was examined by western blot. ***p < 0.001 vs. control; ###*p* < 0.001 vs. BLM

Moreover, we also found obvious cellular senescence in the BLM group according to SA-β-gal staining, indicating that BLM provoked senescence in lung tissue and quercetin obviously attenuated cellular senescence of lung tissues (Fig. 2A), which further verified the reduced protein expression of p16 and p21 following quercetin treatment in pulmonary fibrosis rats (Fig. 2B).

Furthermore, loss of PTEN has been evidenced to be a critical inducer of lung fibrosis (Tian et al. 2019). Therefore, to explore the potential regulatory mechanism of quercetin, PTEN and PTEN-mediated PI3K/AKT signaling were examined. As shown in Fig. 2C, in BLM-induced pulmonary fibrosis rats, the protein expression of PTEN in lung tissues was greatly reduced, while protein expression of p-PI3K and p-AKT was significantly elevated; however, these changes were partly reversed by quercetin administration.

### Quercetin inhibits BLM-stimulated senescence of alveolar epithelial cells, lessens SASP cytokine secretion, and regulates PETN/PI3K/AKT signaling

**Fig. 3:**
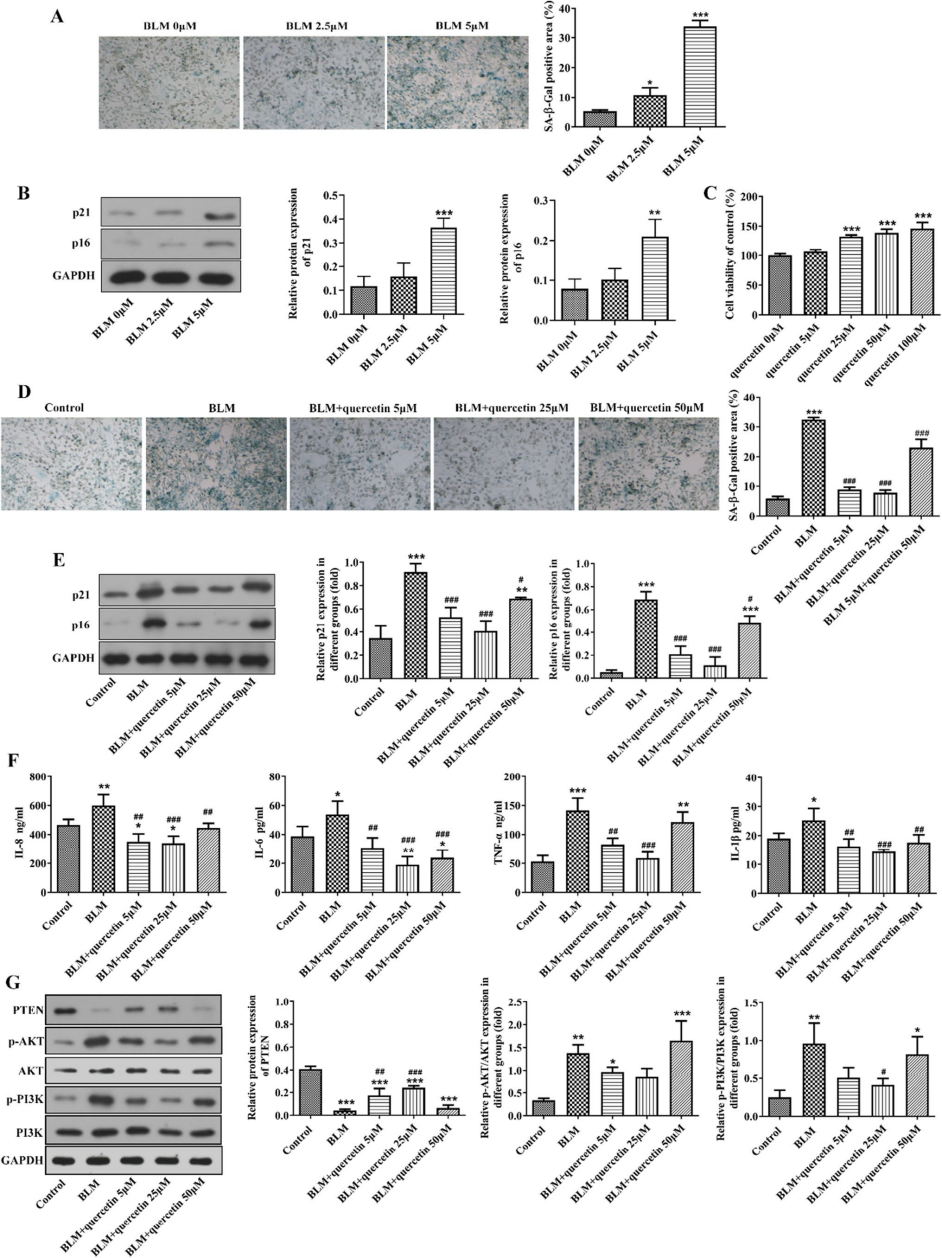
Quercetin inhibits BLM-stimulated senescence of alveolar epithelial cells, lessens SASP cytokine secretion, and regulates PETN/PI3K/AKT signaling. **A** Alveolar epithelial RLE-6TN cells were stimulated by BLM, and SA-β-gal staining was performed to assess cellular senescence. **B** The protein expression of p21 and p16 was examined by western blot. ***p < 0.001 vs. BLM 0 μM. **C** RLE-6TN cells were treated with increasing concentrations (5, 25, 50, and 100 μM) of quercetin, and CCK-8 assay was conducted to determine cell viability. ***p < 0.001 vs. quercetin 0 μM. **D** RLE-6TN cells were pre-treated with quercetin prior to BLM stimulation, and SA-β-gal staining was performed to assess cellular senescence. **E** The protein expression of p21 and p16 was examined by western blot. **F** The production of IL-8, IL-6, IL-1β, and TNF-α in the culture medium from RLE-6TN cells was detected. **G** The protein expression of PTEN, p-PI3K, PI3K, p-AKT, and AKT in RLE-6TN cells was examined by western blot. *p < 0.05, **p < 0.01, and ***p < 0.001 vs. control; #*p* < 0.05, ##*p* < 0.01, and ###*p* < 0.001 vs. BLM

Subsequently, to verify whether quercetin regulates senescence in alveolar epithelial cells, BLM was adopted to stimulate RLE-6TN cells. As shown in Figs. 3A, B and 5 μM of BLM greatly increased SA-β-gal-positive cells and the protein expression of p21 and p16, suggesting that BLM triggered alveolar epithelial senescence. RLE-6TN cells were treated with increasing concentrations (5, 25, 50, and 100 μM) of quercetin, and CCK-8 assay revealed that quercetin treatment was positive for cell viability (Fig. 3C). Next, RLE-6TN cells were pre-treated with quercetin prior to BLM stimulation to explore the effect of quercetin on alveolar epithelial cell senescence. It was discovered from Fig. 3D, E that quercetin treatment greatly restricted BLM-triggered SA-β-gal-positive cells and protein expression of p21 and p16, suggesting that quercetin could prevent BLM-induced alveolar epithelial cell senescence. In addition, we also examined the SASP cytokine secretion of RLE-6TN cells, and the results revealed that BLM greatly promoted the production of IL-8, IL-6, IL-1β, and TNF-α in the culture medium from RLE-6TN cells, while quercetin treatment greatly hindered their production (Fig. 3F). Furthermore, consistent with in vivo findings, the in vitro experiments also revealed the inhibitory effects of quercetin on BLM-downregulated PTEN expression and BLM-upregulated p-PI3K (Fig. 3G).

### Quercetin ameliorated collagen deposition in fibroblasts

**Fig. 4:**
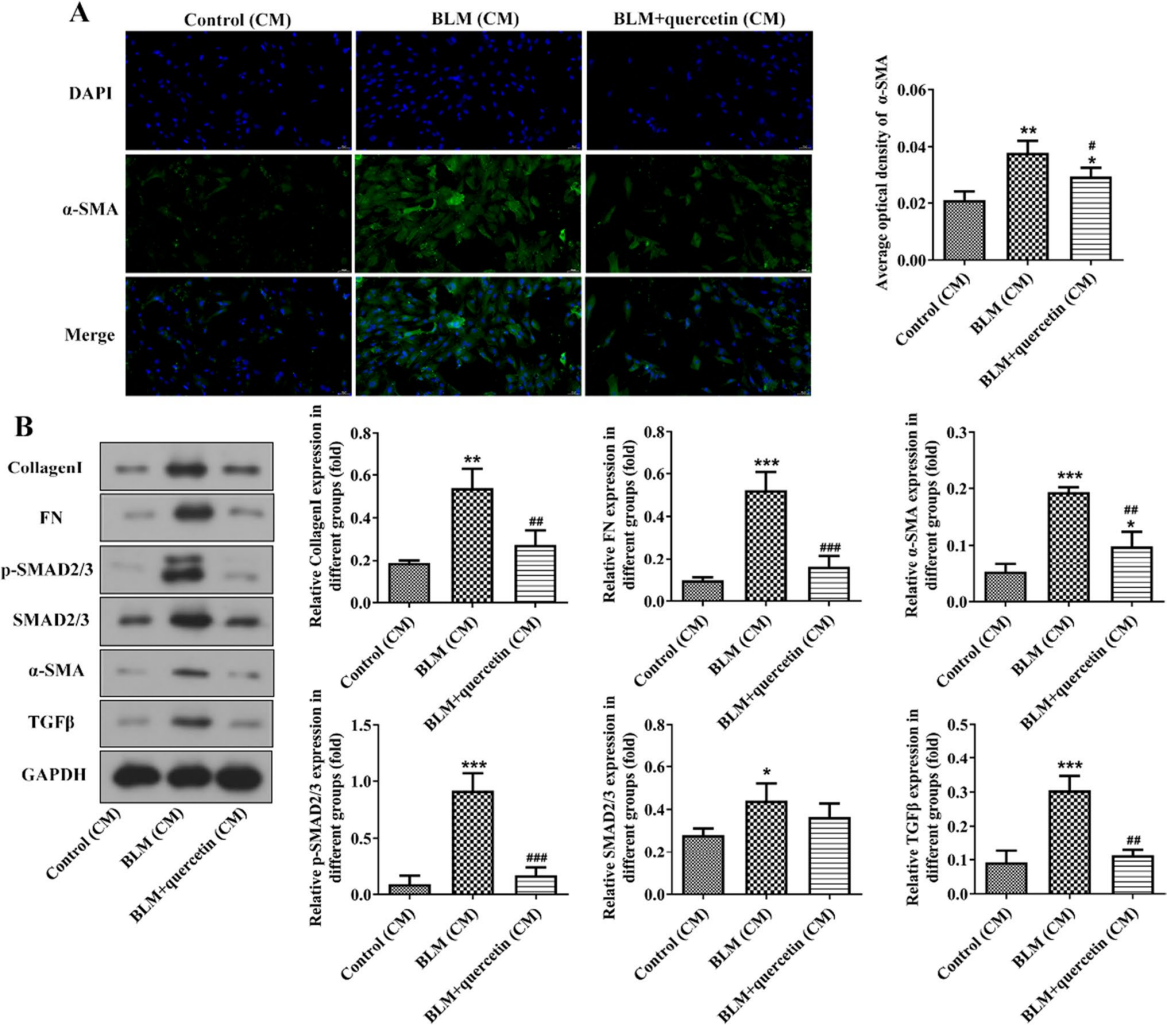
Quercetin ameliorated collagen deposition in fibroblasts. **A** CCC-REPF-1 fibroblasts were exposed to RLE-6TN-derived CM from each group for 3 days, and the α-SMA level was examined by immunofluorescence. **B** Protein expression of collagen I, FN, α-SMA, TGF-β, and p-SMAD2/3 in CCC-REPF-1 cells was examined by western blot. *p < 0.05, **p < 0.01, and ***p < 0.001 vs. control (CM); #*p* < 0.05, ##*p* < 0.01, and ###*p* < 0.001 vs. BLM (CM)

Next, CCC-REPF-1 fibroblasts were exposed to RLE-6TN-derived CM from each group for 3 days, and the elevated α-SMA level was discovered in BLM (CM)-stimulated CCC-REPF-1 cells in comparison to control (CM)-cultured CCC-REPF-1 cells, whereas BLM+quercetin (CM) greatly repressed α-SMA level compared to BLM (CM) (Fig. 4A). In addition, the results from western blot revealed that the protein expression of collagen I, FN, α-SMA, TGF-β, and p-SMAD2/3 in CCC-REPF-1 cells was greatly increased when cultured with BLM (CM), which was remarkably abolished when cultured with BLM+quercetin (CM) (Fig. 4B), confirming that alveolar epithelial cell senescence–secreted SASP exerted pro-fibrotic effects in fibroblasts and quercetin could alleviate senescent cell–mediated collagen deposition in fibroblasts.

### PTEN knockdown weakens the inhibitory effects of quercetin on BLM-stimulated senescence of alveolar epithelial cells and collagen deposition in fibroblasts

**Fig. 5:**
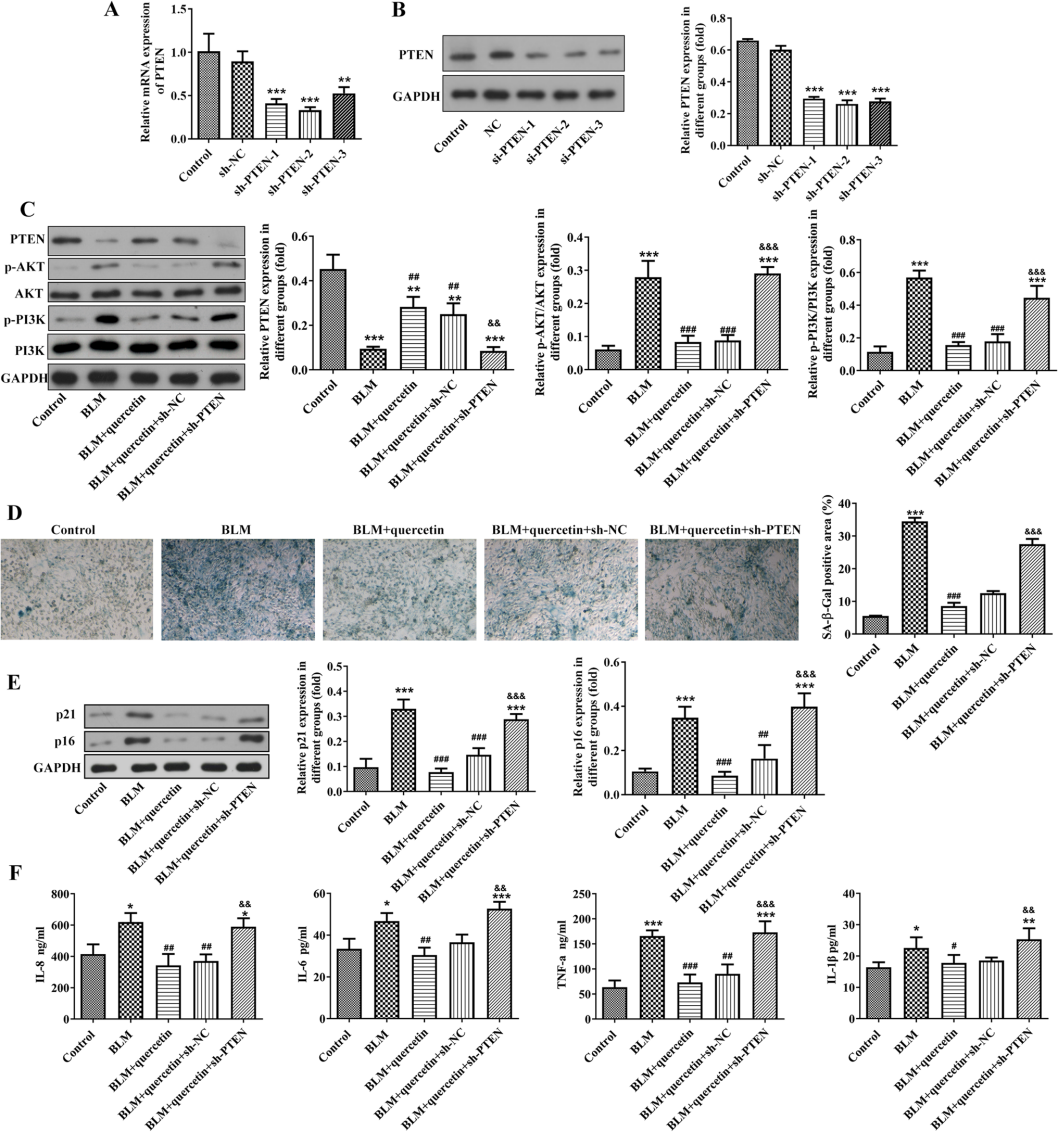
PTEN knockdown weakens the inhibitory effects of quercetin on BLM-stimulated senescence of alveolar epithelial cells. **A** RLE-6TN cells were transfected with sh-PTEN-1/2/3 or sh-NC, and the mRNA level of PTEN was examined by qRT-PCR. **B** The protein expression of PTEN was examined by western blot. **p < 0.01, ***p < 0.001, vs. sh-NC. **C** sh-PTEN-transfected RLE-6TN cells were treated with 25 μM of quercetin prior to BLM stimulation, and the expression level of PETN/PI3K/AKT signaling–related proteins was examined by western blot. **D** SA-β-gal staining was performed to assess cellular senescence. **E** The protein expression of p21 and p16 was examined by western blot. **F** The production of IL-8, IL-6, IL-1β, and TNF-α in the culture medium from RLE-6TN cells was detected. **p < 0.01 and ***p < 0.001 vs. control; #*p* < 0.05, ##*p* < 0.01, and ###*p* < 0.001 vs. BLM; &*p* < 0.05, &&*p* < 0.01, and &&&*p* < 0.001 vs. BLM+quercetin+sh-NC

**Fig. 6:**
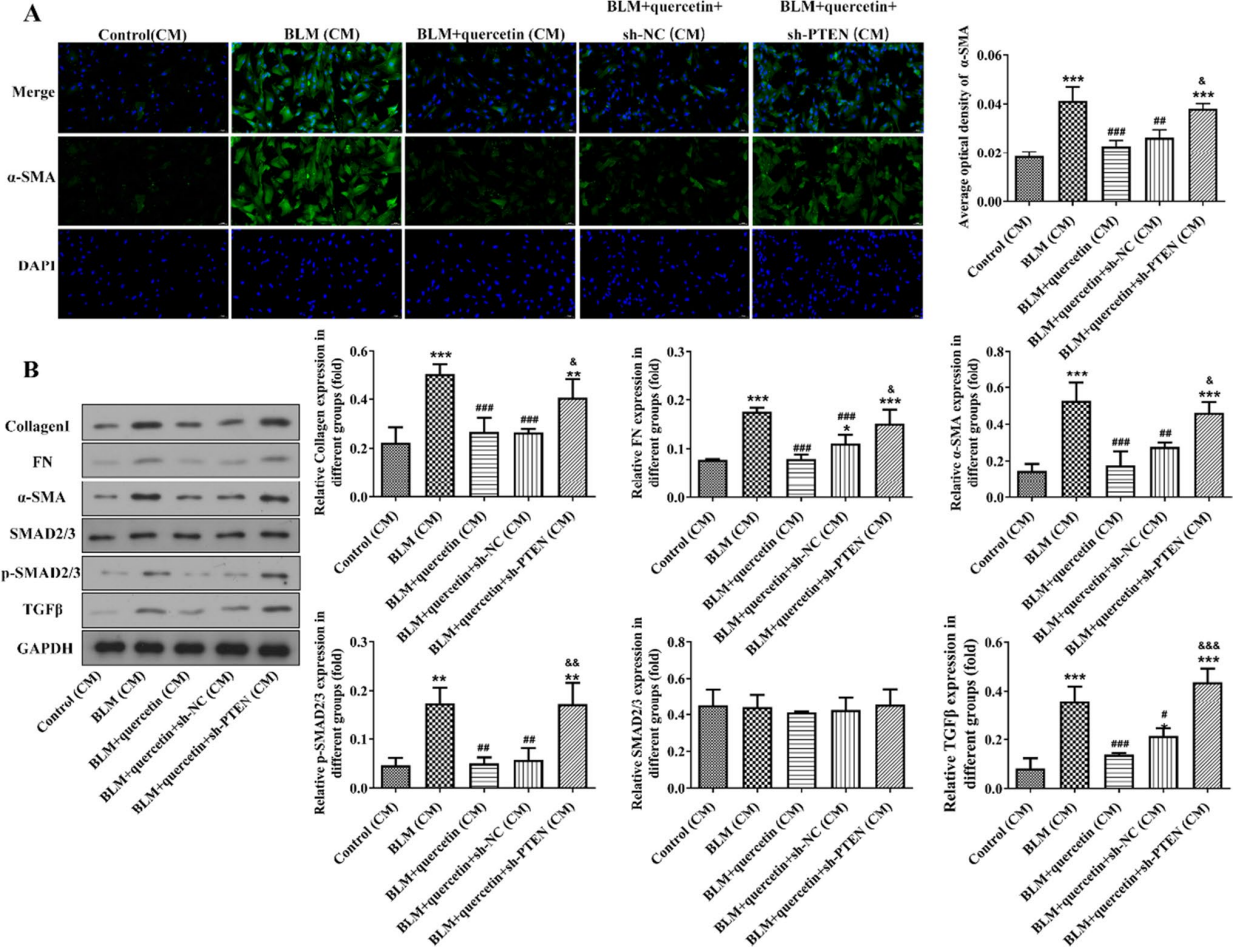
PTEN knockdown weakens the inhibitory effect of quercetin on BLM-stimulated collagen deposition in fibroblasts. **A** CCC-REPF-1 fibroblasts were exposed to RLE-6TN-derived CM from each group for 3 days, and the α-SMA level was examined by immunofluorescence. **B** Protein expression of collagen I, FN, α-SMA, TGF-β, and p-SMAD2/3 in CCC-REPF-1 cells was examined by western blot. Data were presented as mean ± SD. *p < 0.05, **p < 0.01, and ***p < 0.001 vs. control (CM); #*p* < 0.05, ##*p* < 0.01, and ###*p* < 0.001 vs. BLM (CM); &*p* < 0.05, &&*p* < 0.01, and &&&*p* < 0.001 vs. BLM+quercetin+sh-NC (CM)

Next, to verify the indispensable role of PTEN behind the anti-profibrotic activity of quercetin, the loss-of-function experiment was conducted by transfection with sh-PTEN-1/2/3 in RLE-6TN cells. Compared to the sh-NC group, the mRNA level and protein expression of PTEN were greatly reduced in the sh-PTEN-1/2/3 groups (Fig. 5A-B). Then, sh-PTEN-2 was used for subsequent experiments, and the sh-PTEN-transfected RLE-6TN cells were treated with 25 μM of quercetin prior to BLM stimulation. It was found that PTEN knockdown greatly weakened the impacts of quercetin on PETN/PI3K/AKT signaling (Fig. 5C). In addition, the inhibitory effects of quercetin on BLM-induced SA-β-gal-positive cells, protein expression of p21 and p16, and SASP cytokine production in RLE-6TN cells were significantly abolished by PTEN knockdown (Fig. 5D, F). Furthermore, CCC-REPF-1 fibroblasts were exposed to culture medium from above different groups, and the results from Fig. 6A, B revealed that compared to the BLM+quercetin+sh-NC (CM) group, no matter the α-SMA fluorescence intensity, or the protein expression of collagen I, FN, α-SMA, TGF-β, and p-SMAD2/3 was greatly enhanced in the BLM+quercetin+sh-PTEN (CM) group, proving that PTEN knockdown partly restricted the inhibitory effect of quercetin on collagen deposition in fibroblasts.

### Quercetin regulates intestinal dysbacteriosis in BLM-induced pulmonary fibrosis rats

**Fig. 7:**
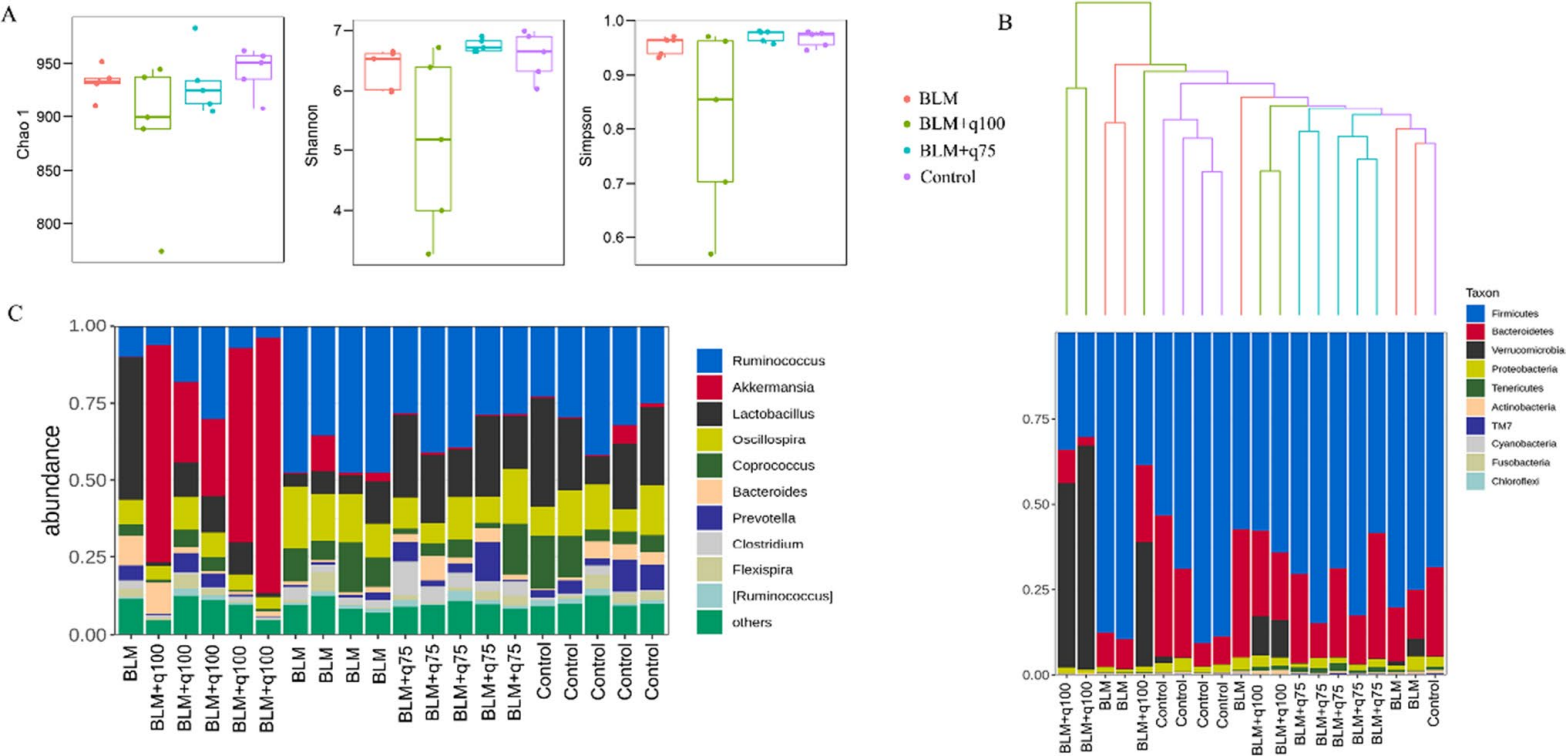
Quercetin regulates intestinal dysbacteriosis in BLM-induced pulmonary fibrosis rats. **A** Chao1 index, Shannon index, and Simpson index of each group. **B** β-diversity of gut microbiota. **C** The abundance of gut microbiota at the genus level. BLM+q75 indicates BLM+quercetin (75 mg/kg); BLM+q100 indicates BLM+quercetin (100 mg/kg)

Eventually, we explored the effects of quercetin on BLM-caused intestinal dysbacteriosis in rats. At first, we assessed the microbial diversity and richness (Chao1 index, Shannon index, and Simpson index) among groups. As shown in Fig. 7A, there was no significant difference in the Chao1 index, while the BLM+q100 group exhibited a relatively low level of Shannon and Simpson indexes, meaning a reduced α-diversity of the gut microbial community in pulmonary fibrosis rats after the administration of quercetin (100 mg/kg). In addition, the β-diversity of gut microbiota of each group was evaluated using UPGMA cluster tree, and the clustering results revealed that the dominant bacteria in gut were *Firmicutes*, *Bacteroidetes*, and *Verrucomicrobia*, and the samples of the BLM group were distant from those of the control group, whereas the samples of the BLM+q100 group were distinct from other groups (Fig. 7B). At the genus level, the top ten abundant bacteria were *Ruminococcus*, *Akkermansia*, *Lactobacillus*, *Oscillospira*, *Coprococcus*, *Bacteroides*, *Prevotella*, *Clostridium*, *Flexispira*, and [*Ruminococcus*] (Fig. 7C). It was notable that the abundance of *Akkermansia* was extremely boosted in the BLM+q100 group, compared to other groups.

## Discussion

Pulmonary fibrosis is a chronic progressive disease of unknown causes with high mortality and limited treatment options. In-depth understanding of the pathogenesis and search for effective drugs are urgent for the prevention and treatment of pulmonary fibrosis. Quercetin has shown outstanding anti-fibrotic activity in different diseases (Hohmann et al. 2019; Lu et al. 2018; Wu et al. 2017). In the present study, we explored the effects and potential mechanisms of quercetin on pulmonary fibrosis in vivo and in vitro. In BLM-induced pulmonary fibrosis rats, quercetin exerted its protective effects by reducing histological injury and collagen deposition, lessening cellular senescence, and regulating gut microbiota. In BLM-induced alveolar epithelial cell senescence, quercetin inhibited senescence, lessened SASP cytokine secretion of alveolar epithelial cells, and further ameliorated collagen deposition in fibroblasts. In addition, quercetin might exert its functional effects by regulating the PTEN/PI3K/AKT signaling pathway. Our findings provide an in-depth understanding of the potential mechanism behind the protective role of quercetin against pulmonary fibrosis.

There is growing evidence supporting the causative role of senescence in the development and progression of pulmonary fibrosis. The major cell types involved in pulmonary fibrosis are fibroblasts and alveolar epithelial cells, and cellular senescence is regarded as a major driver of pulmonary fibrosis (Merkt et al. 2020; Waters et al. 2018; Yao et al. 2021). Cellular senescence markers were widely distributed within lung tissues of pulmonary fibrosis, and the deletion of senescent cells could rejuvenate pulmonary health in aged mice (Hashimoto et al. 2016). Additionally, it is reported by Guan et al. that BMP4 could inhibit cellular senescence in lung fibroblasts to attenuate fibroblast-to-myofibroblast differentiation and extracellular matrix production. BMP4 deficiency–mediated cellular senescence of fibroblasts promotes fibrosis in the lung (Guan et al. 2022). Therefore, targeting senescent cells is therapeutic in moderating lung fibrosis, providing an alternative approach for drug discovery and verification. The anti-fibrotic activity of quercetin against pulmonary fibrosis has been confirmed in previous studies, and in terms of its regulatory mechanism, Hohmann et al. demonstrated that quercetin could restore the susceptibility of senescent fibroblasts to proapoptotic stimuli and relieve BLM-induced pulmonary fibrosis in aged mice, suggesting quercetin as a viable option to prevent fibroblast senescence (Hohmann et al. 2019). However, except fibroblasts, whether quercetin regulates senescence in alveolar epithelial cells needs to be verified. In this respect, we adopted BLM to induce senescence in alveolar epithelial cells. Quercetin not only reduced the number of senescent cells and inhibited the expression of p21 and p16, the classical senescence markers, but also restricted the production of SASP, evidenced by the reduced IL-8, IL-6, IL-β, and TNF-α levels, demonstrating that quercetin inhibited cellular senescence in alveolar epithelial cells. Moreover, fibroblasts were exposed to quercetin-adjusted SASP from alveolar epithelial cells, and results suggested that quercetin-adjusted SASP significantly retard collagen deposition in fibroblasts, thereby alleviating pulmonary fibrosis. Hence, quercetin might hinder the progression of pulmonary fibrosis by ameliorating the senescence of alveolar epithelial cells.

The gut microbiota is a sophisticated micro-ecosystem with diverse and plentiful bacteria modulating the immune system, host metabolism, and tissue homeostasis (Budden et al. 2017; Dang and Marsland 2019). The cross-talk between the gut microbiota and the lungs, called the gut-lung axis, uncovers a regulatory function and feedback of gut microbiota on lung disorders (Shi et al. 2021; Chioma et al. 2021). Targeting gut microbiota homeostasis or specific members of gut communities can modulate the progression of pulmonary fibrosis (Zhang et al. 2023; Hu et al. 2023; Wu et al. 2022). Up to date, accumulating documents have confirmed the beneficial activity of quercetin in modulating gut microbiota to ameliorate different diseases such as osteoarthritis and diarrhea (Lan et al. 2021; Xu et al. 2021; Shi et al. 2020); however, whether quercetin can regulate gut microbiota to alleviate pulmonary fibrosis remains unclear. In this study, we for the first time explored the effects of quercetin on gut microbiota in BLM-induced pulmonary fibrosis rats. Here, it was interesting to find that the feces samples of BLM-induced pulmonary fibrosis rats with quercetin (100 mg/kg) treatment exhibited extreme changes in microbial diversity and bacteria abundance, as the microbial diversity (Shannon and Simpson indexes) was dramatically reduced and the *Akkermansia* abundance was dramatically boosted. *Akkermansia muciniphila* is an oval-shaped, non-motile, strictly anaerobic, and gram-negative bacterium that colonizes the intestinal mucosa of humans and rodents and is regarded as a next-generation probiotic (Zhang et al. 2019). Recently, emerging evidence reveals the therapeutic potential of *Akkermansia* in multiple diseases, including inflammatory bowel disease, obesity, diabetes, and nonalcoholic fatty liver disease (Rodrigues et al. 2022; Han et al. 2022). It has been reported that *Akkermansia* is reversely correlated with inflammation and fibrosis, and *Akkermansia* supplementation has been proven to ameliorate chronic renal interstitial fibrosis and liver fibrosis, indicating an anti-fibrosis property of *Akkermansia* (Yamamoto et al. 2021; Pei et al. 2023; Keshavarz Azizi Raftar et al. 2021); however, whether *Akkermansia* is also beneficial to alleviate pulmonary fibrosis remains unclear. In this study, quercetin at 100 mg/kg exhibited an outstanding efficiency in attenuating pulmonary fibrosis in in vivo experiments and remarkably boosted the abundance of *Akkermansia*, suggesting that the high abundance of *Akkermansia* caused by quercetin at 100 mg/kg might partly account for the anti-fibrotic activity of quercetin against pulmonary fibrosis.

However, there were some limitations in this study. Firstly, we did not examine the SASP cytokines in the animal model, which might be actually closer to what would happen in patients with pulmonary fibrosis. Secondly, due to limited experimental expenditure and time, we conducted the animal experiments only using female rats, while utilization of both male and female rats would be beneficial to further validate these findings. Thirdly, this study provided a preliminary illustration about quercetin-mediated intestinal dysbacteriosis against pulmonary fibrosis; however, to demonstrate the critical role of *Akkermansia*, further experiments such as administration of *Akkermansia* alone in pulmonary fibrosis animal model were required. These limitations deserve to be explored in our future work.

## Conclusion

Taken together, quercetin can alleviate the progression of BLM-induced pulmonary fibrosis through retarding alveolar epithelial cell senescence by regulating the PTEN/PI3K/AKT signaling pathway. In addition, quercetin also influences intestinal dysbacteriosis in pulmonary fibrosis. Quercetin may be a promising candidate for the clinical prevention and treatment of pulmonary fibrosis.

## Data Availability

The data supporting the conclusions of this manuscript will be made available from the corresponding author on reasonable request.
